# Study on the Mechanisms of Glrα3 in Pain Sensitization of Endometriosis

**DOI:** 10.3390/ijms25158143

**Published:** 2024-07-26

**Authors:** Peiya Fan, Rong Liu, Yan Li, Shixuan Wang, Tian Li

**Affiliations:** 1 Department of Obstetrics and Gynecology, National Clinical Research Center for Obstetrics and Gynecology, Tongji Hospital, Tongji Medical College, Huazhong University of Science and Technology, Wuhan 430000, China; m202176199@hust.edu.cn (P.F.); liurongdoctor@163.com (R.L.); liyan@tjh.tjmu.edu.cn (Y.L.); sxwang@tjh.tjmu.edu.cn (S.W.); 2 Key Laboratory of Cancer Invasion and Metastasis (Ministry of Education), Hubei Key Laboratory of Tumor Invasion and Metastasis, Tongji Hospital, Tongji Medical College, Huazhong University of Science and Technology, Wuhan 430000, China

**Keywords:** endometriosis, Glrα3, pain, central sensitization

## Abstract

Endometriosis, often associated with chronic pelvic pain, can lead to anxiety and depression. This study investigates the role and mechanism of Glycine receptor alpha 3 (Glrα3) in the central sensitization of pain in endometriosis, aiming to identify new therapeutic targets. Using a Glrα3 knockout mouse model of endometriosis, we employed behavioral tests, qPCR, immunofluorescence, Nissl staining, MRI, and Western blot to assess the involvement of Glrα3 in central pain sensitization. Our results indicate that endometriosis-induced hyperalgesia and anxiety–depressive-like behaviors are linked to increased Glrα3 expression. Chronic pain in endometriosis leads to gray matter changes in the sensory and insular cortices, with Glrα3 playing a significant role. The inhibition of Glrα3 alleviates pain, reduces neuronal abnormalities, and decreases glial cell activation. The absence of Glrα3 effectively regulates the central sensitization of pain in endometriosis by inhibiting glial cell activation and maintaining neuronal stability. This study offers new therapeutic avenues for the clinical treatment of endometriosis-related pain.

## 1. Introduction

Endometriosis is a prevalent inflammatory gynecological disorder that not only causes chronic pelvic pain but also frequently leads to infertility, significantly impacting patients’ quality of life and economic status [1,2]. The persistence of pain in endometriosis, associated with the stage, location, number, size, and severity of lesions, remains poorly understood and may continue even after surgical interventions [3]. This enduring pain is closely linked to increased inflammatory sensitivity in the peripheral nervous system and alterations in pain processing within the central nervous system, potentially leading to neuroplastic changes that cause heightened pain responses to minimal stimuli [4].

Recent studies suggest a shared etiology between the overlapping conditions of chronic pain and mood disorders in patients with endometriosis, which may relate to changes in the central pathways of pain processing, known as central sensitization [5]. Central sensitization is characterized by increased responsiveness to low-threshold stimuli, enhanced neuronal membrane excitability, increased synaptic efficacy, reduced inhibitory neuron activity, and altered synaptic plasticity [6,7]. These pain-induced changes in brain regions can be detected via MRI techniques, revealing alterations in functional connectivity, gray matter volume, cortical surface area, and thickness [8,9,10]. However, research on pain-induced brain changes in endometriosis remains limited.

The glycine receptor alpha 3 (Glrα3) component plays a crucial role in regulating inflammatory pain hypersensitivity [11]. In models of inflammatory pain, animals with Glrα3 gene mutations or deletions exhibit either an absence of pain hypersensitivity or a rapid recovery from pain sensitization [12,13]. Furthermore, increased expression levels of Glrα3 in the insular region of mouse models of endometriosis are associated with pain hypersensitivity [14], suggesting that targeting Glrα3 could offer new directions for treating chronic pain induced by endometriosis.

In addition to behavioral and MRI assessments, changes at the neuronal functional level can be further pinpointed through various staining techniques. Nissl staining reflects neuronal functional activity [15], and c-Fos is widely used to track pain stimulus transmission pathways and locate neurons [16]. Recent findings highlight the significant role of neuroglial cells in maintaining and developing pain. Activated neuroglial cells influence synaptic plasticity in the central nervous system by modulating neuronal activity [17], thereby triggering and exacerbating pain sensitivity through the release of pro-inflammatory cytokines and chemokines such as TNF-α, IL-6, and IL-1β [18]. Increases in astrocytes and microglia have been observed in the dorsal horn of the spinal cord in mouse models of endometriosis-associated pain [19]. Moreover, women with endometriosis are at a high risk of developing psychological disorders such as anxiety and depression [20]. Neuroglial cells are also implicated in depression and anxiety, where microglia contribute to the onset and progression of these conditions through the activation of the NLRP3 inflammasome and the overproduction of inflammatory cytokines [21,22].

Thus, a precise analysis of neuronal and neuroglial changes in brain regions associated with endometriosis pain is crucial for understanding the mechanisms of central sensitization. This understanding could pave the way for future approaches targeting Glrα3 as a novel analgesic for endometriosis.

## 2. Results

### 2.1. Endometriosis Induces Hypersensitivity to Pain and Anxiety–Depressive Behaviors

**Figure 1:**
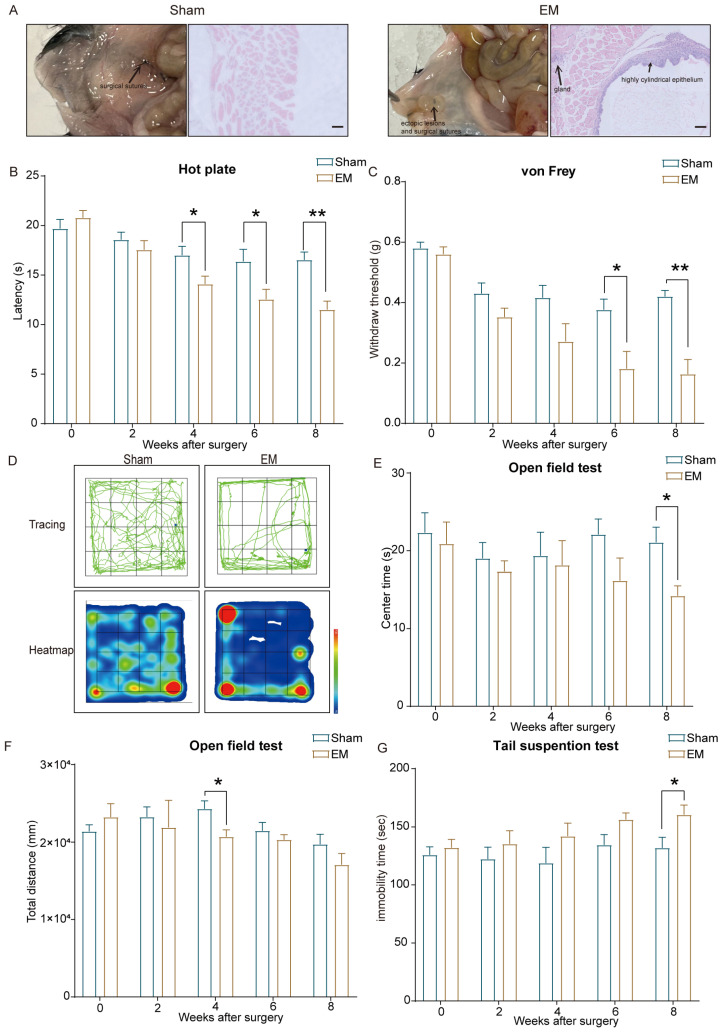
Behavioral test results. (**A**) Macroscopic and histological images of the ventral abdominal wall in two groups of mice (scale bar: 200 µm). H&E staining of endometriotic-like lesions to confirm the success of the endometriosis model; the typical endometrioid gland, including highly cylindrical epithelium, could be seen. (**B**) Changes in thermal withdrawal latency (TWL) at weeks 4, 6, and 8 post-surgery, showing reduced TWL in the EM group compared to the Sham group. (**C**) Reductions in mechanical withdrawal threshold (MWT) in the EM group at weeks 6 and 8 post-surgery compared to the Sham group. (**D**) Representative activity tracks in the open field test at week 8 post-surgery for EM and Sham group mice. (**E**) Reduced central area activity time in the EM group compared to the Sham group in the open field test at week 8 post-surgery. (**F**) Reduced total distance traveled in the open field test by the EM group compared to the Sham group at week 4 post-surgery. (**G**) Increased immobility time in the tail suspension test for the EM group at week 8 post-surgery compared to the Sham group. * *p* < 0.05, ** *p* < 0.01. n = 5 per group.

Experimental outcomes demonstrated that EM group mice developed typical vesicular lesions, cyst-like structures, and gland-like formations, which were absent in the Sham group. Both macroscopic and histological examinations confirmed the successful establishment of the endometriosis model (Figure 1A).

Hot plate test results indicated no significant differences in thermal withdrawal latency (TWL) between the EM and the Sham group mice before surgery. From the fourth week post-surgery, TWL was significantly reduced in the EM group compared to the Sham group, a trend that persisted until the eighth week (Figure 1B). The von Frey test revealed no pre-surgical differences in mechanical withdrawal thresholds (MWT) between groups. From the sixth week post-surgery, MWT was significantly lower in the EM group compared to the Sham group, continuing through the eighth week (Figure 1C).

In the open field test, the movement trajectory of mice in the EM group in the central area was significantly shorter than that in the Sham group (Figure 1D), and the exploration time of mice in the EM group in the central area was shorter than that in the Sham group (Figure 1E). In the total distance of the open field test, the EM group mice showed a decrease compared with the Sham group in the 4th week (Figure 1F). The tail suspension test revealed that by the eighth week post-surgery, immobility time was significantly longer in the EM group compared to the Sham group (Figure 1G).

The significant reductions in TWL and MWT, along with decreased central area activity time and increased immobility time, indicate heightened pain sensitivity and anxiety–depressive behaviors in the EM group.

### 2.2. Upregulation of Glrα3 in the S1 and IC Brain Regions of Mice with Endometriosis

**Figure 2:**
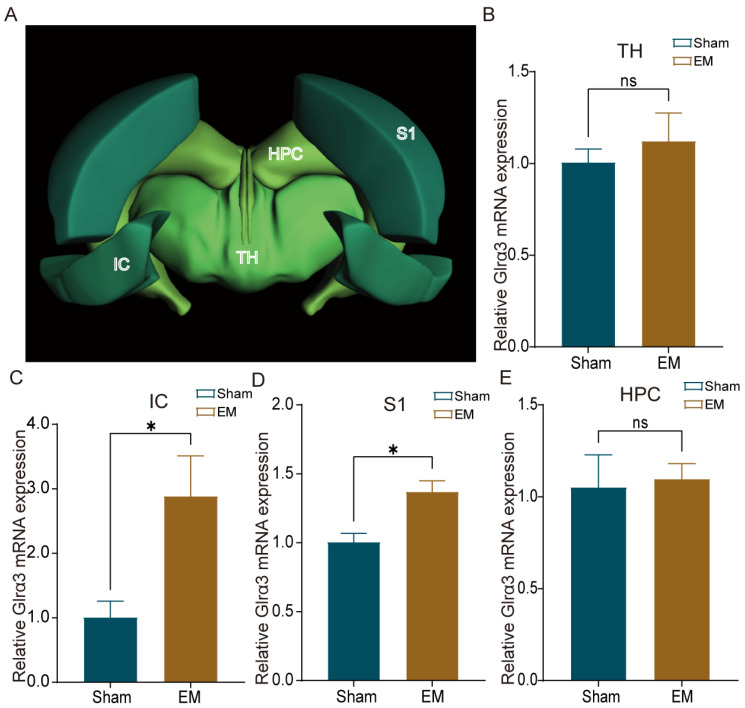
Differential expression of Glrα3 mRNA in brain regions. (**A**) Schematic of spatial localization in the brain regions IC, S1, HPC, and TH. (**B**) No significant difference in Glrα3 expression in the TH region between the two groups. (**C**) Increased expression of Glrα3 in the IC region of the EM group mice. (**D**) Increased expression of Glrα3 in the S1 region of the EM group mice. (**E**) No significant differences in Glrα3 expression in the HPC region between the groups. * *p* < 0.05, ns indicates *p* > 0.05. n = 4/5 per group.

qPCR was utilized to assess the expression of Glrα3 in brain regions associated with pain and emotion processing. The insular cortex (IC) and primary somatosensory cortex (S1) are critical in pain processing and emotional response, while the hippocampus (HPC) is associated with pain memory, and the thalamus (TH) serves as a relay for pain signals. qPCR results indicated that Glrα3 mRNA levels were significantly elevated in the IC and S1 regions of EM group mice compared to Sham, with no significant differences detected in the HPC and TH regions (Figure 2A–E).

### 2.3. Inhibition of Glrα3 Alleviates Pain Hypersensitivity and Reduces Anxiety–Depressive Behaviors

**Figure 3:**
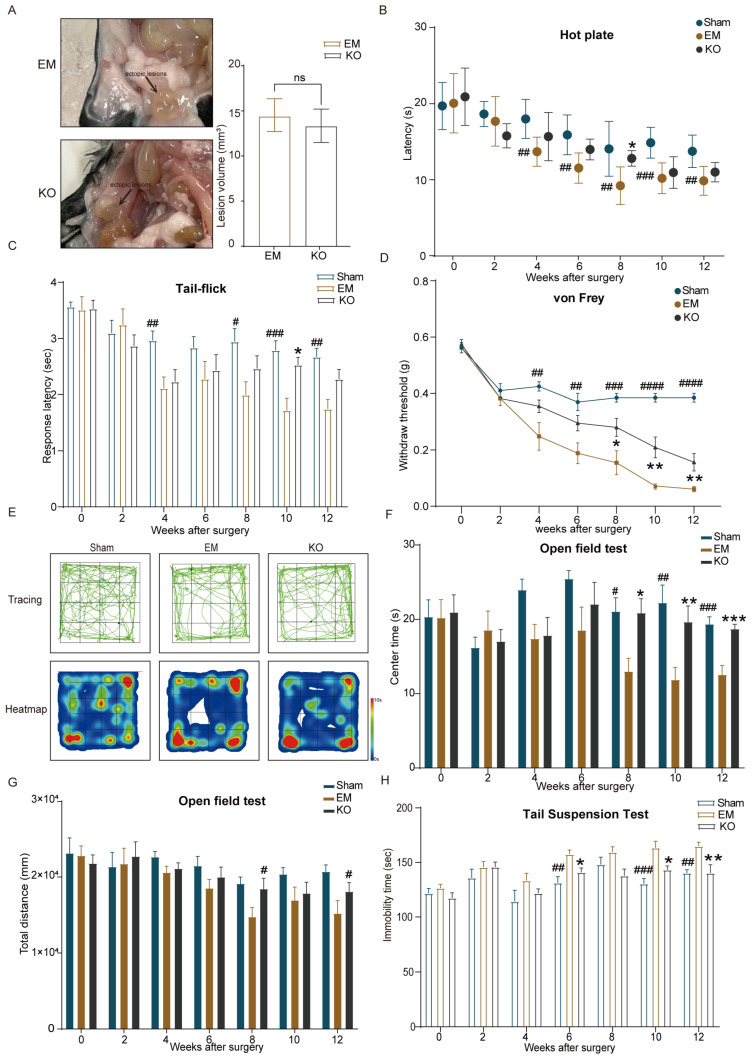
Behavioral test results. (**A**) No significant difference in lesion volume between EM and KO groups. (**B**) Changes in TWL detected in the hot plate test post-surgery across three groups. (**C**) Changes in TWL in the tail-flick test post-surgery across three groups. (**D**) Changes in MWT detected in the von Frey test post-surgery across three groups. (**E**) Representative activity tracks in the open field at week 12 post-surgery for three groups. (**F**) Changes in central activity time in the open field test post-surgery across three groups. (**G**) Changes in total activity distance in the open field test post-surgery across three groups. (**H**) Changes in immobility time in the tail suspension test post-surgery across three groups. Comparisons: * EM vs. KO, ^#^ EM vs. Sham, with significance levels * *p* < 0.05, ** *p* < 0.01, *** *p* < 0.001, ^#^ *p* < 0.05, ^##^ *p* < 0.01, ^###^ *p* < 0.001, ^####^ *p* < 0.001. n = 8 per group.

The hot plate and tail-flick tests showed that thermal pain sensitivity was significantly higher in the EM group than in the Sham group after surgery, while inhibiting Glrα3 in the KO group significantly decreased pain sensitivity by the sixth week and the eighth week post-surgery (Figure 3A,B). The von Frey test indicated an increase in mechanical pain sensitivity in the EM group from the fourth week post-surgery. In contrast, mechanical pain sensitivity in the KO group showed recovery from the eighth to the twelfth week post-surgery.

The open field test revealed a reduction in central area activity time starting from the eighth week post-surgery in the EM group, while the KO group exhibited extended central area activity time compared to the EM group (Figure 3C–F). The tail suspension test indicated prolonged immobility time in the EM group during the sixth week, tenth week, and twelfth week post-surgery, whereas the KO group exhibited shorter immobility times during later stages of the experiment (Figure 3G).

These results suggest that the observed reductions in thermal and mechanical pain sensitivity, as well as improvements in anxiety–depressive behaviors in the KO group, are likely due to the inhibition of Glrα3, which mitigates the hypersensitivity and emotional disturbances induced by endometriosis.

### 2.4. Inhibition of Glrα3 Ameliorates Reductions in Gray Matter Volume in the S1 and IC Regions

**Figure 4:**
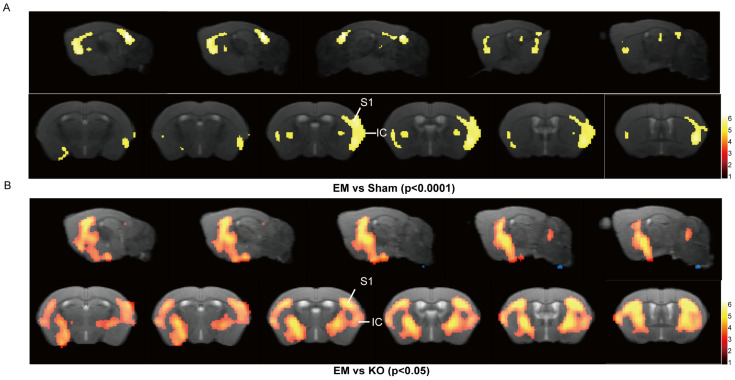
VBM analysis differences. (**A**) Voxel comparison of gray matter volume (GMV) between EM and Sham groups. (**B**) Voxel comparison of GMV between EM and KO groups. The color bar’s top and bottom numbers indicate the t values of the statistical results. n = 8 per group.

Previous studies have shown that pain can lead to reductions in gray matter volume in specific brain regions [23]. In this study, we employed voxel-based morphometry (VBM) to compare changes across three groups of mice, identifying significant clusters using a threshold of *p* < 0.0001 for EM versus Sham comparisons, and *p* < 0.05 for EM versus KO comparisons. The results indicated that, compared to Sham, EM mice exhibited reduced gray matter volume (GMV) in the sensory cortex and insula. Furthermore, compared to KO mice, EM mice showed reductions in GMV in the sensory cortex, amygdala, thalamus, and insular cortex (Table 1, Figure 4A,B).

The reductions in gray matter volume (GMV) observed in the sensory and insular cortex of EM mice are indicative of pain-induced neuroanatomical changes specific to endometriosis. The amelioration of GMV reductions in the KO group implies that Glrα3 inhibition has a neuroprotective effect, potentially reversing pain-related brain alterations rather than merely reflecting differences in overall brain morphology.

### 2.5. Inhibition of Glrα3 Alleviates Neuronal Alterations in the IC and S1 Regions

To further focus on the effects of endometriosis on brain regions, guided by MRI and preliminary qPCR results, this study specifically examined the S1 sensory cortex and insula. 

**Figure 5:**
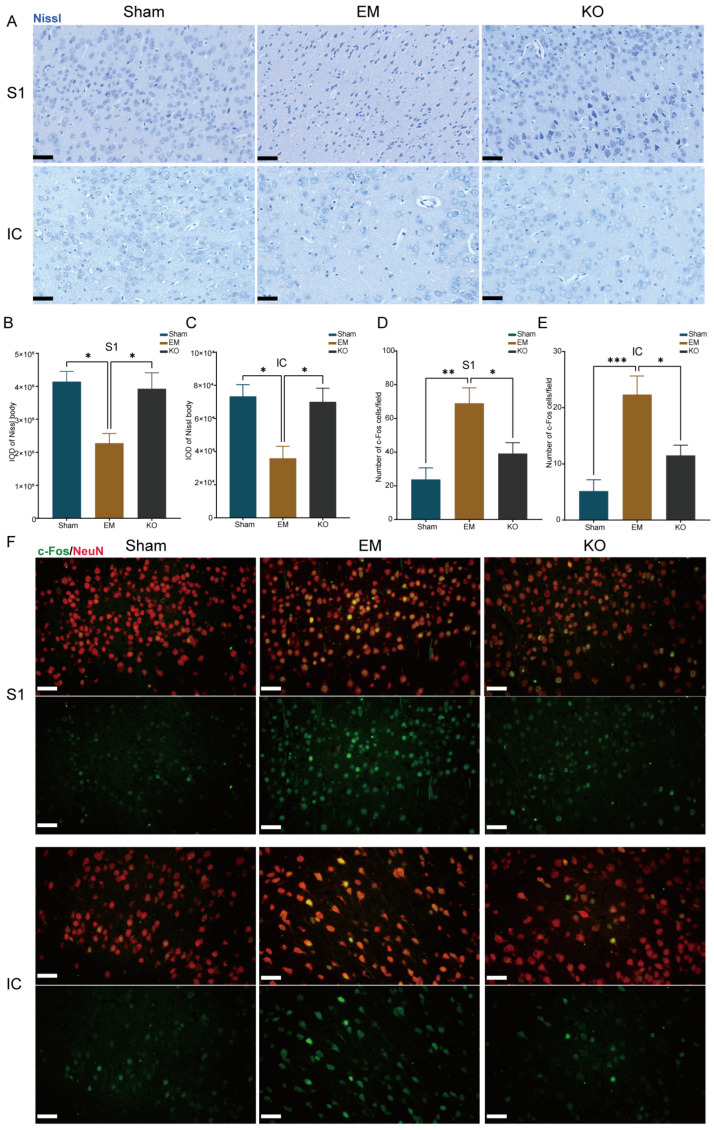
Differences in neuronal activity in the S1 and IC brain regions among three groups. (**A**) Representative Nissl-stained sections from the S1 and IC brain regions of three groups, scale bar 50 µm. (**B**) Nissl staining results in the S1 region. (**C**) Nissl staining results in the IC region. (**D**) Statistical results of Neun/c-Fos co-staining in the S1 region. (**E**) Statistical results of Neun/c-Fos co-staining in the IC region. (**F**) Representative sections of Neun/c-Fos co-staining in the S1 and IC brain regions, scale bar 50 µm. * *p* < 0.05, ** *p* < 0.01, *** *p* < 0.001. IOD (Integrated Optical Density). n = 4 per group.

Nissl staining was used to observe changes in Nissl bodies, crucial components of neuronal protein synthesis, which significantly decrease when neurons are damaged. The results revealed notable alterations in neuronal structures in EM mice; in the S1 and IC regions, the integrated optical density of Nissl staining was significantly reduced compared to Sham. In contrast, KO mice exhibited an increase in total optical density compared to EM mice (Figure 5A–C). Additionally, c-fos staining was utilized to reflect neuronal activity [24]. The results showed a significant increase in NeuN+/c-fos+ cells in the S1 and IC regions in EM mice compared to Sham; this increase was reduced in KO mice compared to EM (Figure 5D–F). 

### 2.6. Inhibition of Glrα3 Reduces Activation of Glial Cells in the IC and S1 Regions

Previous research has suggested that the increased activation of glial cells can exacerbate pain sensitivity [25]. To explore whether microglia and astrocytes were also more activated in the IC and S1 cortex, this study employed various techniques for detection. 

**Figure 6:**
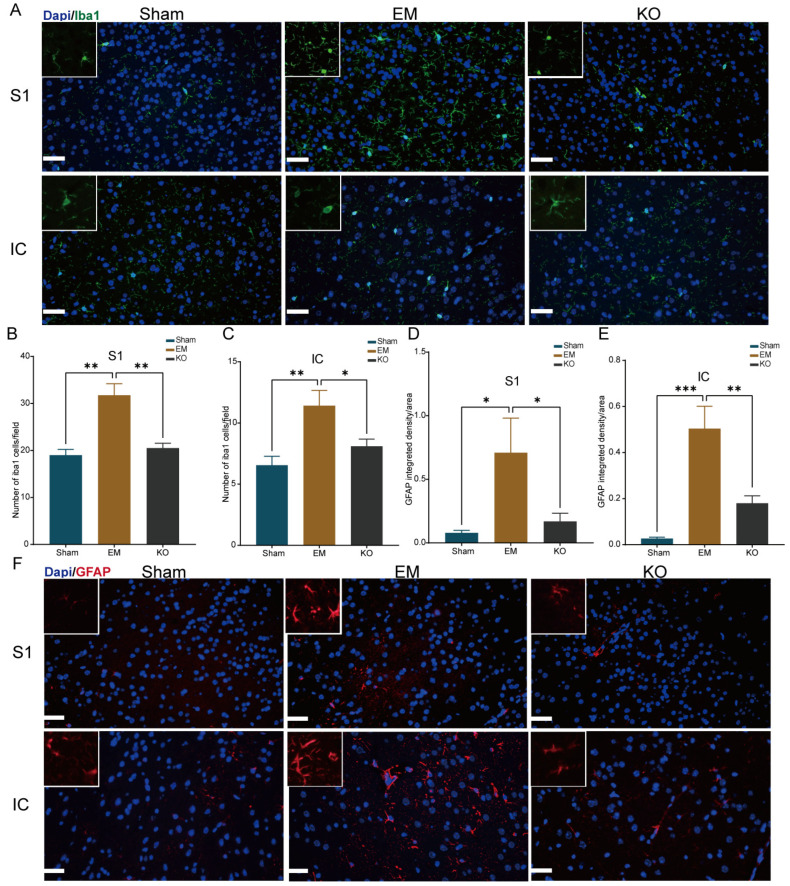
Activation of glial cells in the S1 and IC brain regions among three groups. (**A**) Representative Iba1-stained sections from the S1 and IC brain regions, scale bar 50 µm. (**B**) Iba1 staining results in the S1 region. (**C**) Iba1 staining results in the IC region. (**D**) GFAP staining results in the S1 region. (**E**) GFAP staining results in the IC region. (**F**) Representative GFAP-stained sections from the S1 and IC brain regions, scale bar 50 µm. * *p* < 0.05, ** *p* < 0.01, *** *p* < 0.001. n = 4 per group.

The immunofluorescence results indicated that the activation of microglia (labeled with Iba1) and astrocytes (labeled with GFAP) was significantly higher in EM mice compared to Sham. This activation was significantly reduced in the KO group compared to EM (Figure 6A–F). 

**Figure 7:**
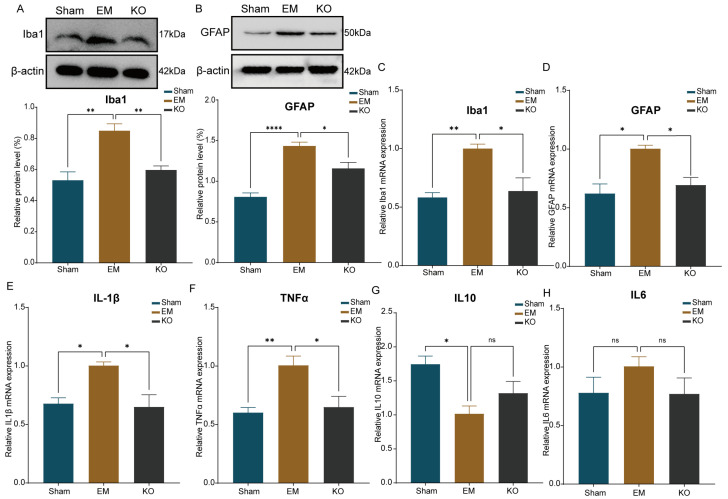
Quantification of glial cells and inflammatory factors expression. (**A**) Protein blot analysis for Iba1 across three groups. (**B**) Protein blot analysis for GFAP across three groups. (**C**) Quantification of Iba1 mRNA levels in three groups. (**D**) Quantification of GFAP mRNA levels in three groups. (**E**) Quantification of IL1β mRNA levels in three groups. (**F**) Quantification of TNFα mRNA levels in three groups. (**G**) Quantification of IL10 mRNA levels in three groups. (**H**) Quantification of IL6 mRNA levels in three groups. * *p* < 0.05, ** *p* < 0.01, **** *p* < 0.0001. ns: no significance. n = 4 per group.

Western blotting (WB) and quantitative PCR (qPCR) further validated these findings (Figure 7A–D). Additionally, the study examined inflammatory factors associated with glial cells. qPCR results showed significant increases in the pro-inflammatory factors TNF-α and IL-1β in EM mice compared to Sham, with significant reductions in these factors in KO mice compared to EM. There were no statistically significant differences in IL-6 expression among the groups. The expression of the anti-inflammatory factor IL-10 was reduced in EM mice compared to Sham (Figure 7E–H).

## 3. Discussion

Endometriosis is defined as the presence of endometrial-like tissue outside the uterus [26]. The growth of ectopic endometrial tissue is accompanied by an increased secretion of cytokines, angiogenic factors, and neurotrophic factors. These factors collectively promote inflammation, neovascularization, and heightened neuronal sensitivity, contributing to the pain associated with endometriosis [27]. Treatment options for endometriosis-related pain are diverse, including surgical interventions, pharmacological treatments (analgesics, hormones, and non-hormonal therapies), and non-pharmacological strategies [28]. However, because of the complexity of endometriosis, the recurrence of pain after stopping treatment is common [29]. Therefore, a deeper understanding of the pain mechanisms in endometriosis, particularly the role of central sensitization, is crucial for developing more effective pain management strategies.

In this study, we employed a murine model of endometriosis and used behavioral, imaging, and molecular biology techniques to investigate the mechanisms underlying central sensitization to pain in endometriosis. Our findings indicate that, between the fourth week and the sixth week post-surgery, mice began to exhibit lowered pain thresholds. By the eighth week, mice displayed behaviors indicative of anxiety and depression. These results suggest that the peritoneal model of endometriosis can effectively mimic the clinical manifestations observed in some endometriosis patients, namely reduced pain thresholds [3] and accompanying symptoms of anxiety and depression [30].

Previous research has highlighted that hypersensitivity to pain in endometriosis is associated with an increased expression of Glrα3 in the insular cortex of mice [14]. Other studies on pain mechanisms have demonstrated that the knockout of Glrα3 mitigates the inflammatory pain hypersensitivity induced by complete Freund’s adjuvant [31]. Our preliminary qPCR screenings suggest that Glrα3 predominantly affects the S1 and IC, implicating these regions in the mediation of pain in endometriosis. Behavioral assessments in Glrα3-deficient mice have shown that the absence of this receptor can alleviate pain hypersensitivity and anxiety–depressive behaviors in the later stages of the disease. Moreover, thermal pain primarily activates the outer laminae I and II of the spinal cord, whereas mechanical pain predominantly triggers the inner lamina II neurons, explaining the observed differences in the timing of thermal and mechanical pain regulation in our studies [32]. Notably, the staging and volume of endometriotic lesions do not directly correlate with the intensity of pain, consistent with our findings [3].

Neuroimaging can detect resting-state functional connectivity, gray matter density, and the levels of GABA and Glx (glutamate and glutamine), thereby predicting or reflecting the degree of pain sensitivity [33]. Women with endometriosis-associated chronic pelvic pain exhibit enhanced connectivity in the somatosensory cortex and the persistent activation of the somatosensory pain pathway [34]. We used structural magnetic resonance imaging to examine the effects of endometriosis-related pain on brain gray matter. Pain intensity correlates with gray matter volume in various cortical (insula, medial frontal gyrus, temporal gyrus, anterior cingulate cortex) and subcortical (hippocampus, thalamus, amygdala) regions [35], and prolonged pain duration is associated with cortical reorganization and gray matter loss [36]. Our findings indicate that the reduced gray matter in areas such as the sensory and insular cortex in endometriosis model mice may reflect the consequences of chronic nociceptive transmission. The deletion of the glra3 gene appears to induce a partially reversible increase in gray matter volume in regions including the sensory cortex, insula, amygdala, and thalamus. These results align with recent clinical findings that greater gray matter volume is associated with longer pain tolerance [37]. However, the underlying cellular and molecular mechanisms of gray matter changes remain unclear. Potential changes involving glial cells, axonal sprouting, dendritic branching and synaptogenesis, neurogenesis, and angiogenesis, possibly due to increased nociceptive input and similar to learning-related plasticity, might be involved [38]. Therefore, molecular biological assessments were conducted in the relevant brain regions to further understand the reasons behind the differences in these abnormal brain regions.

In this study, we examined neuronal changes in the S1 and IC of endometriosis model mice, employing Nissl and c-Fos staining to assess the alterations. We observed a significant reduction in Nissl bodies and an increase in c-fos expression in both regions in the EM group mice, suggesting pronounced neuronal alterations that correlate with the pain mechanisms previously identified in bone cancer pain research, where pain is closely related to neuronal functional changes and neuronal damage is indicated by a reduction in Nissl bodies [39]. Restoring Nissl bodies in the hippocampus has been shown to provide analgesic and antidepressant effects [40]. An upregulation of neuronal c-Fos is considered indicative of increased pain signal transmission [41]. These findings suggest that endometriosis induces structural and functional abnormalities in the neurons within the S1 and IC, implicating Glrα3 in the modulation of pain associated with endometriosis by potentially stabilizing neuronal integrity in these critical areas.

Glial cells are known to perpetuate pain sensitivity by prolonging the release of inflammatory mediators, thereby sensitizing neighboring central projection neurons and inducing central sensitization, ultimately leading to chronic pain [42]. Glial cells also regulate neuronal activity through the provision of metabolic substrates and modulation of synaptic components [43]. Our study observed the activation of microglia and astrocytes in the S1 and IC regions of endometriosis model mice. The secretion of pro-inflammatory cytokines by pro-inflammatory microglia, such as IL-1β and TNF-α, is recognized as a key driver of central sensitization [44]. Changes in the expression of inward-rectifying potassium channels in astrocytes can influence neuronal discharge patterns, contributing to the development of pain hypersensitivity [45]. Our analyses further revealed that the balance between pro-inflammatory and anti-inflammatory factors was disrupted in these regions, suggesting that the absence of Glrα3 could mitigate pain hypersensitivity in endometriosis by reducing the activation of glial cells and the release of inflammatory factors in the S1 and IC.

Nevertheless, this study has certain limitations. In our behavioral testing, we will optimize the modeling method to further reduce the interference of the surgery itself on the behavioral results, and appropriately increase the detection experiments for local pain sensitivity. This will make our experimental results more reliable and accurate. In the mechanistic studies, we may have overlooked changes in brain regions at other time points post-modeling and have not dynamically and deeply explored the alterations in neural circuits. Although the mouse model has provided valuable insights, the physiological and pathological differences between this model and the human disease may limit the direct applicability of our findings. In future studies, we will strive to address these limitations to enhance the relevance and translational potential of our research.

## 4. Materials and Methods

### 4.1. Animals

SPF-grade C57BL/6J mice, 8–10 weeks old, were purchased from Hubei Bente Biotechnology Co., Ltd. (Wuhan, China); Glrα3 gene knockout mice of the same age were purchased from Jiangsu Jicui Yaokang Biotechnology Co., Ltd. (Nanjing, China). These mice were housed and bred at the Animal Center of the Research Building, Tongji Hospital, Huazhong University of Science and Technology. The housing conditions were a temperature of 25 °C with sufficient food and water, and a 12 h light/12 h dark cycle. All procedures and housing were conducted in accordance with the standardized guidelines of the Experimental Animal Center of Tongji Hospital and were approved and supervised by the Ethics Committee of Tongji Hospital (Ethics Approval Number: TJH-202303041).

### 4.2. Methods

#### 4.2.1. Animal Model of Endometriosis

The model of endometriosis mouse was established according to the method described in our previous study [46]. Donor mice were euthanized, their uterine horns excised, and placed in phosphate-buffered saline (PBS). Each horn was divided into three equal parts. Uterine biopsies were sutured to the peritoneum using a 7-0 vicryl thread. The same surgical method was applied to the control mice (Sham), with the exception that no endometrial tissue was introduced; sutures were affixed to the peritoneum.

A comprehensive examination of the peritoneum and visceral organs was conducted using an anatomical microscope to identify any endometriosis lesions. Specimen lesions suspected to be significant were meticulously gathered and initially preserved in 10% neutral-buffered formalin for a day, and subsequently stored in 70% ethanol. To confirm endometriosis, visible lesions underwent Hematoxylin and Eosin (H&E) staining to verify the presence of endometrial glands and stroma. Mice without endometriosis were excluded from further analysis. The volume of endometriosis lesions was measured using calipers.

#### 4.2.2. Behavioral Testing

The mechanical paw withdrawal threshold (PWT) test was performed using von Frey fibers according to previous reports [47]. Mice were acclimated for at least 30 min in Plexiglass chambers placed on a mesh platform. Calibrated von Frey monofilaments (0.04, 0.07, 0.16, 0.4, 0.6, 1.0, 1.4, and 2.0 g of pressure, in ascending order) were applied perpendicularly to the central part of the plantar area of the hind paw. The filament needs to be kept bent for 5 s or until a withdrawal reflex is evoked. Sudden paw retraction, shaking, or licking were considered as positive responses. An interval of at least 30 s should be maintained between stimuli to avoid sensitization. The withdrawal threshold for each animal was calculated using the up-and-down method.

Thermal hyperalgesia was measured using the hot plate test and the hot tail-flick test. Mice were placed on the plate for 30 min to acclimate to the environment. The hot plate was maintained at 52 °C, with a cutoff time of 20 s. The response was measured when the mouse rapidly withdrew any paw from the surface (flicking, licking), other than walking. To avoid tissue harm, mice were promptly taken off the hot plate following a response indicative of pain sensation [48]. In the hot tail-flick test, mice were gently placed in a rodent restrainer, exposing their tails. The tail’s tip (1–2 cm) was immersed in water at a temperature of 52 °C. The duration taken for the animal to retract its tail from the water was timed. To mitigate the risk of tissue damage, a shorter cut-off time of 10 s was utilized [49].

Open-field test (OFT) reflects anxiety-like behavior based on thigmotaxis in mice [50]. Following an acclimation period of approximately one hour in the laboratory, each mouse was individually placed in the center of a square enclosure (40 × 40 × 40 cm). A video camera mounted above the enclosure captured the mice’s activity. Their behavior was monitored for a total of 5 min using a computerized video tracking system running the VisuTrack program (Novsoft, Shanghai, China). The total distance of the open field experiment reflects the activity ability of mice. The distance and time at center regions were recorded and compared which was used to determine anxiety. Anxious mice are expected to spend less time exploring the central area and leave fewer trajectories in the central area.

The tail suspension test assesses depressive behavior in mice by observing their immobility while suspended, a condition from which they cannot escape [51]. After a 1 h acclimation period in the experimental environment, each mouse was suspended 90 cm above the floor using adhesive tape placed approximately 1 cm from the tip of the tail. Initially, the mice exhibited escape attempts, but these behaviors diminished over time, leading to periods of immobility. The immobility time was recorded using a stopwatch for the final 4 min of the 6 min test. Each mouse was tested individually to avoid interference from other animals. Increased immobility during the tail suspension test is indicative of greater depressive behavior.

#### 4.2.3. MRI Image Acquisition and Processing

Animal MRI examinations were conducted using a 9.4 T small animal MRI scanner (Bruker 94/20, Ettlingen, Germany) at the Center for Molecular Imaging and Translational Medicine, Xiamen University. All mice were positioned in an extended prone position and administered a 1.5% isoflurane/oxygen mixture. T2-weighted images were utilized in this study, as T1 tissue contrast between mouse gray and white matter is less distinct at high magnetic field strengths. MRI images were acquired with the following parameters: RARE factor = 8, echo time = 33 ms, field of view = 35 × 35 mm, image size = 256 × 256, slice thickness = 1 mm, matrix size = 256 × 256, flip angle = 90 degrees, encompassing 35 contiguous slices. The total MRI scanning time per mouse was approximately 10 min.

MRI image analysis was conducted using the Statistical Parametric Mapping 12 toolkit, spmmouseIHEP toolkit, and custom software written in MATLAB R2017b. Initially, each T2-weighted image was aligned with the mouse template in the spmmouseIHEP to the third ventricle and magnified 10-fold to accommodate the difference in total brain volume between humans and rodents. Following this, each image underwent segmentation to create probabilistic delineations of gray matter (GM), white matter, and cerebrospinal fluid. This process utilized tissue probability maps available in the spmmouseIHEP toolkit and then normalized to Paxinos and Franklin’s space using exponential Lie algebra for diffeomorphic anatomical registration. Transformation matrices were used to modulate the normalized gray matter images. The gray matter images were then smoothed using a Gaussian kernel with an 8 mm full-width at half-maximum. These modulated images were subsequently utilized for voxel-based morphometry (VBM) analysis [52].

The voxel-based morphometry (VBM) analysis was performed on images smoothed using the general linear model in the spmmouseIHEP toolkit. Voxels from regions of interest (ROI) were extracted for further quantitative analysis. A t-test was utilized to determine differences between groups. Notably, the differences between the EM and Sham groups were more pronounced, with significant regions identified at a voxel threshold of *p* < 0.0001. Meanwhile, significant differences between the EM and KO groups were identified at a voxel threshold of *p* < 0.05.

#### 4.2.4. Western Blot

The method was conducted as previously described [53]. Simply put, the primary somatosensory cortex (S1) and the insular cortex (IC) of the mouse brain are dissected and mixed for protein extraction. Protein quantification was achieved using the Coomassie Brilliant Blue G250, with a standard curve for calibration. The protein samples were then separated using 12.5% SDS-PAGE and subsequently transferred onto polyvinylidene fluoride (PVDF) membranes. The membranes were blocked with 5% milk for 1.5 h, followed by overnight incubation at 4 °C with primary antibodies. The next day, after TBST washing, the membranes were exposed to secondary antibodies at a temperature of 37 °C for an hour. Protein bands were then visualized through the ChemiDoc TMXRS+ system and analyzed with Image LabTM software Version 4.1 (Bio-Rad, Hercules, CA, USA). Each experiment was independently repeated three times. Primary antibodies included IBA1 (ABclonal, Wuhan, China, A19776, 1:2000) and GFAP (Abcam, Cambridge, UK, ab7260, 1:8000), while the secondary antibody was HRP Goat Anti-Rabbit IgG (H+L) (ABclonal, AS014, 1:5000).

#### 4.2.5. Immunofluorescence Staining

The method was slightly modified from previous experiments [54]. Mice were perfused with 0.1 mol/L PBS followed by 4% PFA. The brains were then immersed in 4% PFA for 24 h and transferred to 30% sucrose solution until sunk. Brain sections were prepared using a cryostat (Thermo Fisher Scientific, Waltham, MA, USA). After rewarming, washing with PBS, permeabilization with 0.3% Triton X-100, and blocking (QuickBlock™ Blocking Buffer, Beyotime, Nantong, China), the sections were incubated overnight at 4 °C with primary antibodies. This was followed by three washes with PBS and incubation at room temperature with appropriate secondary antibodies for 1 h. After three more PBS washes, the sections were incubated with corresponding secondary antibodies for 1 h at room temperature. The following secondary antibodies were diluted to 1:300 before use. After staining, the tissue sections were incubated with DAPI for 15 min. The sections were then washed for 30 min with PBS and stained with DAPI (1:1000, Sigma Millipore, St. Louis, MO, USA, 1:1000, D9542) to visualize the nuclei. Images were captured using a fluorescence microscope and analyzed with ImageJ software (version 1.54). Primary antibodies included IBA1 (ABclonal, China, A19776, 1:200), GFAP (Abcam, UK, ab7260, 1:200), NeuN (Millipore, USA, 3075598, 1:300), and c-Fos (Abcam, UK, ab214672, 1:200). Secondary antibodies included ABflo^®^ 488-conjugated Goat Anti-Rabbit IgG (H+L) (ABclonal, China, AS053, 1:200), ABflo^®^ 594-conjugated Goat Anti-Rabbit IgG (H+L) (ABclonal, China, AS039, 1:200), and ABflo^®^ 594-conjugated Goat Anti-Mouse IgG (H+L) (ABclonal, China, AS054, 1:200).

#### 4.2.6. Nissl Staining

The method was conducted as previously described [55]. These brain sections were stained with Nissl stain (Sevier, Wuhan, China, G1036). After mounting, images were captured using an upright standard light microscope (OLYMPUS BX53, Tokyo, Japan). The integrated optical density (IOD) of Nissl bodies in the IC and S1 regions was quantified using the Image Pro Plus 6.0 software.

#### 4.2.7. qPCR

The primary somatosensory cortex (S1) and insular cortex (IC) was collected and was preserved in RNAlater solution (Thermo Fisher, Carlsbad, CA, USA). We used Trizol reagent (Thermo Fisher, USA) to extract, and the total RNA and Prime ScriptTM RT reagent Kit (Takara Bio Inc., Shiga, Japan) to reversely transcribe the total RNA. The concentration and purity of each sample was detected by Nanodrop Spectrophotometer (Nanodrop, Wilmington, DE, USA). qPCR was performed by CFX96 Real-Time System (Bio-Rad, USA) using the Fast Start Universal SYBR Green Master kit (Takara Bio Inc., China). Each reaction was performed in triplicate and normalized to β-actin gene expression. Detailed primer sequences are listed in Table S1.

#### 4.2.8. Experimental Design

**Figure 8:**
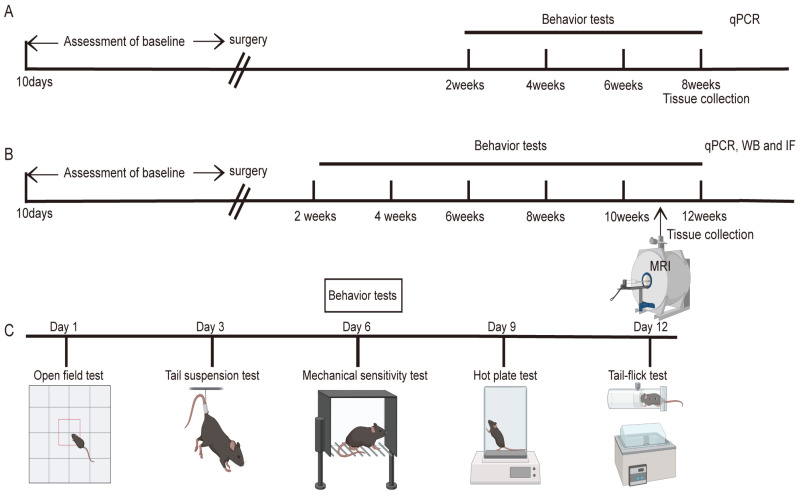
Schematic of the experimental design. (**A**) Schematic workflow for the first batch of experimental mice. (**B**) Schematic workflow for the second batch of experimental mice. (**C**) Detailed timeline for behavioral assessments.

The experiments were conducted in two batches. In the first batch, baseline measurements were taken before the surgery, and behavioral tests were conducted over the course of 8 weeks post-surgery. At the end of week 8, the mice were euthanized, and tissues were collected. In the second batch, baseline measurements were also taken pre-surgery, with behavioral tests conducted throughout the 12 weeks post-surgery. MRI scans were performed during week 11, and at the end of week 12, the mice were euthanized, and tissues were harvested. Notably, the hot tail-flick test was not conducted in the first batch of experiments (Figure 8).

#### 4.2.9. Statistical Analysis

All data are represented using the mean ± standard error of mean (SEM). Univariate analysis of variance (ANOVA) was employed for comparisons across multiple data groups, complemented by Bonferroni-adjusted multiple comparison tests to ensure rigor and accuracy in our analyses. For comparisons between two data sets, we utilized the unpaired two-tailed Student’s *t*-test, a method specifically designed to evaluate significant differences between two groups. Statistical significance was assessed based on *p* values (*p* < 0.05) in GraphPad Prism 9 software.

## 5. Conclusions

Current treatments for endometriosis primarily target ectopic endometrial lesions but fail to directly address pain arising from the central sensitization of the nervous system. Our study demonstrates that the absence of Glrα3 reduces gray matter loss, inhibits glial cell activation, and maintains neuronal stability, thereby regulating central sensitization in endometriosis-associated pain perception. These findings provide new insights into the pain mechanisms of endometriosis and identify novel targets for clinical treatment, potentially leading to more effective and comprehensive management strategies for patients suffering from this condition.

## Figures and Tables

**Table 1:** Comparisons of different brain regions among the Sham and EM and KO groups using structural magnetic resonance imaging.

Brain Region	Voxel	MNI			Peak T Value	Brain Region	Voxel	MNI			Peak T Value
		X (mm)	Y (mm)	Z (mm)				X (mm)	Y (mm)	Z (mm)	
Somatosensory Cortex_left	2461	−2.7555	3.2743	0.9373	5.7204	Caudate Putamen_left	5837	−2.2803	1.8555	−0.0222	2.4598
Temporal Cortex_left	2384	−4.0197	1.2495	−7.9058	6.1893	Caudate Putamen_right	4971	1.9587	−0.2203	−0.6442	2.401
Visual Cortex_left	1984	−2.2701	3.2313	−5.7674	6.2919	Somatosensory Cortex_right	4437	2.3421	−0.7132	−0.1807	2.3139
Auditory Cortex_left	1765	−4.0197	1.2495	−7.9058	6.1893	Somatosensory Cortex_left	4243	−2.2803	1.8713	0.2132	2.5247
Ectorhinal Cortex_left	1616	−4.0107	3.1684	−2.4721	6.1514	Amygdala_right	1719	1.7673	0.078	−0.8753	2.112
Caudate Putamen_left	1456	−2.5611	3.4787	0.9395	5.7985	Insular Cortex_right	1370	2.9181	−1.297	0.5165	2.0951
Amygdala_right	1373	2.3541	1.3608	−0.1547	5.9067	Entorhinal Cortex_right	1358	3.6177	2.8	1.5074	2.5537
Insular Cortex_left	1018	−2.7549	3.378	0.9386	5.7204	Ectorhinal Cortex_left	984	−3.6081	5.6384	−7.2635	2.899
Entorhinal Cortex_left	741	−3.1443	2.4705	−4.9521	5.9972	Piriform Cortex_right	937	2.7249	−1.3019	0.3992	2.1228
Piriform Cortex_right	736	2.3541	1.3608	−0.1547	5.9067	Temporal Cortex_left	922	−3.6081	5.6384	−7.2635	2.22
Hippocampus_right	466	1.6809	1.7318	−2.5008	5.5494	Ectorhinal Cortex_right	801	4.0899	0.9575	1.9538	1.9625
Entorhinal Cortex_right	429	3.4389	4.9995	−2.8159	5.6935	Entorhinal Cortex_left	634	−3.6081	5.6384	−7.2635	2.567
Thalamus_right	422	1.2045	2.8958	−2.2501	5.6191	Insular Cortex_left	605	−3.1641	−1.078	−6.8782	1.9983
Insular Cortex_right	393	3.3039	−1.3672	1.1029	5.2575	Olfactory bulb_right	548	0.1059	−3.3413	−2.7967	2.1406
Subiculum_left	382	−2.3667	3.2249	−5.8849	6.2687	globus pallidus_right	575	1.8639	0.0923	−0.6401	2.401
Dentate gyrus_right	369	1.4799	0.4736	−1.2226	5.5979	Temporal Cortex_right	547	3.8127	2.9027	−1.5493	1.9627
Visual Cortex_right	341	2.1519	−0.1996	−0.2915	5.6628	globus pallidus_left	541	−1.7133	−0.6709	−5.1118	2.2825
Piriform Cortex_left	324	−3.3201	5.3465	−6.9149	5.4311	Primary motor cortex_left	265	−1.9113	−1.5054	−5.2395	2.1345
Caudate Putamen_right	302	3.0417	3.368	0.5748	5.2151	Thalamus_left	153	−1.1319	−0.3214	−4.4029	1.923
Somatosensory Cortex_right	278	3.3039	−1.3672	1.1029	5.2575	Thalamus_right	146	1.2873	0.5645	−1.4563	1.8657
Subiculum_right	235	2.0769	3.5193	−0.4799	5.724	Hippocampus_right	108	2.9547	4.7443	−3.6414	2.2463
Cerebellum_right	199	1.7907	4.1223	−0.8246	1.9713	Hypothalamus_right	58	1.6011	4.7712	−0.4633	2.2093
Retrosplenial cortex_right	178	2.0769	3.5193	−0.4799	5.724	Hippocampus_left	32	−1.8801	3.887	−5.1719	1.792

## Data Availability

Data is contained within the article or Supplementary Materials.

## Supplementary Materials

The following supporting information can be downloaded at: https://www.mdpi.com/article/10.3390/ijms25158143/s1.

## References

[1] Lamvu G., Carrillo J., Ouyang C., Rapkin A. (2021). Chronic Pelvic Pain in Women: A Review. JAMA.

[2] Zondervan K.T., Becker C.M., Missmer S.A. (2020). Endometriosis. N. Engl. J. Med..

[3] Taylor H.S., Kotlyar A.M., Flores V.A. (2021). Endometriosis is a chronic systemic disease: Clinical challenges and novel innovations. Lancet.

[4] Latremoliere A., Woolf C.J. (2009). Central sensitization: A generator of pain hypersensitivity by central neural plasticity. J. Pain.

[5] McNamara H.C., Frawley H.C., Donoghue J.F., Readman E., Healey M., Ellett L., Reddington C., Hicks L.J., Harlow K., Rogers P.A.W. (2021). Peripheral, Central, and Cross Sensitization in Endometriosis-Associated Pain and Comorbid Pain Syndromes. Front. Reprod. Health.

[6] Maddern J., Grundy L., Castro J., Brierley S.M. (2020). Pain in Endometriosis. Front. Cell. Neurosci..

[7] Liang Y., Xie H., Wu J., Liu D., Yao S. (2018). Villainous role of estrogen in macrophage-nerve interaction in endometriosis. Reprod. Biol. Endocrinol..

[8] Qiu D., Wang W., Mei Y., Tang H., Yuan Z., Zhang P., Zhang Y., Yu X., Yang C., Wang Q. (2023). Brain structure and cortical activity changes of new daily persistent headache: Multimodal evidence from MEG/sMRI. J. Headache Pain.

[9] Zheng P., Jia S., Guo D., Chen S., Zhang W., Cheng A., Xie W., Sun G., Leng J., Lang J. (2020). Central Sensitization-Related Changes in Brain Function Activity in a Rat Endometriosis-Associated Pain Model. J. Pain Res..

[10] Terumitsu M., Takado Y., Fukuda K.I., Kato E., Tanaka S. (2022). Neurometabolite Levels and Relevance to Central Sensitization in Chronic Orofacial Pain Patients: A Magnetic Resonance Spectroscopy Study. J. Pain Res..

[11] Zeilhofer H.U., Werynska K., Gingras J., Yévenes G.E. (2021). Glycine Receptors in Spinal Nociceptive Control—An Update. Biomolecules.

[12] Werynska K., Gingras J., Benke D., Scheurer L., Neumann E., Zeilhofer H.U. (2021). A Glra3 phosphodeficient mouse mutant establishes the critical role of protein kinase A-dependent phosphorylation and inhibition of glycine receptors in spinal inflammatory hyperalgesia. Pain.

[13] Harvey R.J., Depner U.B., Wässle H., Ahmadi S., Heindl C., Reinold H., Smart T.G., Harvey K., Schütz B., Abo-Salem O.M. (2004). GlyR alpha3: An essential target for spinal PGE2-mediated inflammatory pain sensitization. Science.

[14] Li T., Mamillapalli R., Ding S., Chang H., Liu Z.W., Gao X.B., Taylor H.S. (2018). Endometriosis alters brain electrophysiology, gene expression and increases pain sensitization, anxiety, and depression in female mice. Biol. Reprod..

[15] Wei Y., Xiao L., Fan W., Zou J., Yang H., Liu B., Ye Y., Wen D., Liao L. (2022). Astrocyte Activation, but not Microglia, Is Associated with the Experimental Mouse Model of Schizophrenia Induced by Chronic Ketamine. J. Mol. Neurosci..

[16] Harris J.A. (1998). Using c-fos as a neural marker of pain. Brain Res. Bull..

[17] Ji R.R., Xu Z.Z., Gao Y.J. (2014). Emerging targets in neuroinflammation-driven chronic pain. Nature reviews. Drug Discov..

[18] Atta A.A., Ibrahim W.W., Mohamed A.F., Abdelkader N.F. (2023). Microglia polarization in nociplastic pain: Mechanisms and perspectives. Inflammopharmacology.

[19] Dodds K.N., Beckett E.A.H., Evans S.F., Hutchinson M.R. (2019). Spinal Glial Adaptations Occur in a Minimally Invasive Mouse Model of Endometriosis: Potential Implications for Lesion Etiology and Persistent Pelvic Pain. Reprod. Sci..

[20] Maulitz L., Stickeler E., Stickel S., Habel U., Tchaikovski S.N., Chechko N. (2022). Endometriosis, psychiatric comorbidities and neuroimaging: Estimating the odds of an endometriosis brain. Front. Neuroendocrinol..

[21] O’Leary L.A., Mechawar N. (2021). Implication of cerebral astrocytes in major depression: A review of fine neuroanatomical evidence in humans. GLIA.

[22] Jia X., Gao Z., Hu H. (2021). Microglia in depression: Current perspectives. Sci. China Life Sci..

[23] Valet M., Gündel H., Sprenger T., Sorg C., Mühlau M., Zimmer C., Henningsen P., Tölle T.R. (2009). Patients with pain disorder show gray-matter loss in pain-processing structures: A voxel-based morphometric study. Psychosom. Med..

[24] Reis C.L.B., Pingueiro-Okada E.M., Luiz K.G., Pedroso G.L., Matsumoto M.A.N., de Menezes L.M., Küchler E.C., Nascimento G.C., Stuani M.B.S. (2023). Orthodontic pain: C-Fos expression in rat brain nuclei after rapid maxillary expansion. J. World Fed. Orthod..

[25] Takeda I., Yoshihara K., Cheung D.L., Kobayashi T., Agetsuma M., Tsuda M., Eto K., Koizumi S., Wake H., Moorhouse A.J. (2022). Controlled activation of cortical astrocytes modulates neuropathic pain-like behaviour. Nat. Commun..

[26] Chapron C., Marcellin L., Borghese B., Santulli P. (2019). Rethinking mechanisms, diagnosis and management of endometriosis. Nat. Rev. Endocrinol..

[27] Song S.Y., Jung Y.W., Shin W., Park M., Lee G.W., Jeong S., An S., Kim K., Ko Y.B., Lee K.H. (2023). Endometriosis-Related Chronic Pelvic Pain. Biomedicines.

[28] Horne A.W., Missmer S.A. (2022). Pathophysiology, diagnosis, and management of endometriosis. BMJ.

[29] Becker C.M., Gattrell W.T., Gude K., Singh S.S. (2017). Reevaluating response and failure of medical treatment of endometriosis: A systematic review. Fertil. Steril..

[30] Gruber T.M., Mechsner S. (2021). Pathogenesis of Endometriosis: The Origin of Pain and Subfertility. Cells.

[31] Harvey V.L., Caley A., Müller U.C., Harvey R.J., Dickenson A.H. (2009). A Selective Role for alpha3 Subunit Glycine Receptors in Inflammatory Pain. Front. Mol. Neurosci..

[32] Targowska-Duda K.M., Peters D., Marcus J.L., Zribi G., Toll L., Ozawa A. (2024). Functional and anatomical analyses of active spinal circuits in a mouse model of chronic pain. Pain.

[33] Niddam D.M., Wang S.J., Tsai S.Y. (2021). Pain sensitivity and the primary sensorimotor cortices: A multimodal neuroimaging study. Pain.

[34] Ferdek M.A., Oosterman J.M., Adamczyk A.K., van Aken M., Woudsma K.J., Peeters B., Nap A., Wyczesany M., van Rijn C.M. (2019). Effective Connectivity of Beta Oscillations in Endometriosis-Related Chronic Pain during rest and Pain-Related Mental Imagery. J. Pain.

[35] Torrecillas-Martínez L., Catena A., O’Valle F., Padial-Molina M., Galindo-Moreno P. (2019). Does experienced pain affects local brain volumes? Insights from a clinical acute pain model. Int. J. Clin. Health Psychol..

[36] Tracey I., Bushnell M.C. (2009). How Neuroimaging Studies Have Challenged Us to Rethink: Is Chronic Pain a Disease?. J. Pain.

[37] Melum T.A., Vangberg T.R., Johnsen L.H., Steingrímsdóttir Ó.A., Stubhaug A., Mathiesen E.B., Nielsen C. (2023). Gray matter volume and pain tolerance in a general population: The Tromsø study. Pain.

[38] Bhatt R.R., Gupta A., Rapkin A., Kilpatrick L.A., Hamadani K., Pazmany E., Van Oudenhove L., Stains J., Aerts L., Enzlin P. (2019). Altered gray matter volume in sensorimotor and thalamic regions associated with pain in localized provoked vulvodynia: A voxel-based morphometry study. Pain.

[39] Yang H.-Y., Zhang F., Cheng M.-L., Wu J., Xie M., Yu L.-Z., Liu L., Xiong J., Zhu H.-L. (2022). Glycogen synthase kinase-3β inhibition decreases inflammation and relieves cancer induced bone pain via reducing Drp1-mediated mitochondrial damage. J. Cell. Mol. Med..

[40] Liu X., Xie Z., Li S., He J., Cao S., Xiao Z. (2021). PRG-1 relieves pain and depressive-like behaviors in rats of bone cancer pain by regulation of dendritic spine in hippocampus. Int. J. Biol. Sci..

[41] Wang J., Yin C., Pan Y., Yang Y., Li W., Ni H., Liu B., Nie H., Xu R., Wei H. (2023). CXCL13 contributes to chronic pain of a mouse model of CRPS-I via CXCR5-mediated NF-κB activation and pro-inflammatory cytokine production in spinal cord dorsal horn. J. Neuroinflamm..

[42] Siouti E., Andreakos E. (2019). The many facets of macrophages in rheumatoid arthritis. Biochem. Pharmacol..

[43] Watkins L.R., Milligan E.D., Maier S.F. (2001). Glial activation: A driving force for pathological pain. Trends Neurosci..

[44] Matejuk A., Ransohoff R.M. (2020). Crosstalk Between Astrocytes and Microglia: An Overview. Front. Immunol..

[45] Ou M., Chen Y., Liu J., Zhang D., Yang Y., Shen J., Miao C., Tang S.J., Liu X., Mulkey D.K. (2023). Spinal astrocytic MeCP2 regulates Kir4.1 for the maintenance of chronic hyperalgesia in neuropathic pain. Prog. Neurobiol..

[46] Jeljeli M., Riccio L.G.C., Chouzenoux S., Moresi F., Toullec L., Doridot L., Nicco C., Bourdon M., Marcellin L., Santulli P. (2020). Macrophage Immune Memory Controls Endometriosis in Mice and Humans. Cell Rep..

[47] de Castro-Jorge L.A., de Carvalho R.V.H., Klein T.M., Hiroki C.H., Lopes A.H., Guimarães R.M., Fumagalli M.J., Floriano V.G., Agostinho M.R., Slhessarenko R.D. (2019). The NLRP3 inflammasome is involved with the pathogenesis of Mayaro virus. PLoS Pathog..

[48] Grim T.W., Schmid C.L., Stahl E.L., Pantouli F., Ho J.H., Acevedo-Canabal A., Kennedy N.M., Cameron M.D., Bannister T.D., Bohn L.M. (2020). A G protein signaling-biased agonist at the μ-opioid receptor reverses morphine tolerance while preventing morphine withdrawal. Neuropsychopharmacology.

[49] Kinsey S.G., Naidu P.S., Cravatt B.F., Dudley D.T., Lichtman A.H. (2011). Fatty acid amide hydrolase blockade attenuates the development of collagen-induced arthritis and related thermal hyperalgesia in mice. Pharmacol. Biochem. Behav..

[50] Barcelon E.E., Cho W.H., Jun S.B., Lee S.J. (2019). Brain Microglial Activation in Chronic Pain-Associated Affective Disorder. Front. Neurosci..

[51] Zeng J., Meng X., Zhou P., Yin Z., Xie Q., Zou H., Shen N., Ye Z., Tang Y. (2019). Interferon-α exacerbates neuropsychiatric phenotypes in lupus-prone mice. Arthritis Res. Ther..

[52] Nie B., Wu D., Liang S., Liu H., Sun X., Li P., Huang Q., Zhang T., Feng T., Ye S. (2019). A stereotaxic MRI template set of mouse brain with fine sub-anatomical delineations: Application to MEMRI studies of 5XFAD mice. Magn. Reson. Imaging.

[53] Guo E., Xiao R., Wu Y., Lu F., Liu C., Yang B., Li X., Fu Y., Wang Z., Li Y. (2022). WEE1 inhibition induces anti-tumor immunity by activating ERV and the dsRNA pathway. J. Exp. Med..

[54] Xie C., Shen X., Xu X., Liu H., Li F., Lu S., Gao Z., Zhang J., Wu Q., Yang D. (2020). Astrocytic YAP Promotes the Formation of Glia Scars and Neural Regeneration after Spinal Cord Injury. J. Neurosci..

[55] Sun D., Gao G., Zhong B., Zhang H., Ding S., Sun Z., Zhang Y., Li W. (2021). NLRP1 inflammasome involves in learning and memory impairments and neuronal damages during aging process in mice. Behav. Brain Funct..

